# Supplementary material for: Immunogenic Properties of MVs Containing Structural Hantaviral Proteins: An Original Study
Source: Pharmaceutics. 2022 Jan 1;14(1):93. doi: 10.3390/pharmaceutics14010093 (PMC8779827; doi:10.3390/pharmaceutics14010093)
Supplement: Supplementary file 1 [file pharmaceutics-14-00093-s001.zip › pharmaceutics-1480287-supplementary.pdf]

pharmaceutics

Supplementary Materials: Immunogenic Properties of MVs Containing Structural Hantaviral Proteins: An Original Study

Layaly Shkair, Ekaterina Evgenevna Garanina, Ekaterina Vladimirovna Martynova *, Alena Igorevna Kolesnikova, Svetlana Sergeevna Arkhipova, Angelina Andreevna Titova, Albert Anatolevich Rizvanov and Svetlana Francevna Khaiboullina

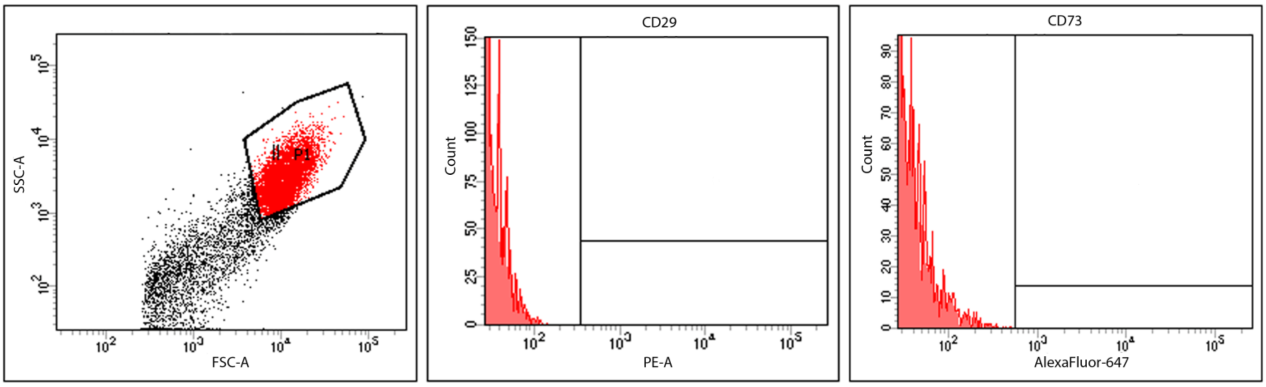

Figure S1. Immunophenotyping analysis of adipose tissue-derived mMSCs by flow cytometry. Non-stained mMSCs were used for gating to reduce autofluorescence. Population of mMSC (P1) was selected according to the cell size and granularity .Cells were analyzed using Flow Cytometry on FACS Aria III (Becton, Dickinson and Company, Becton Drive Franklin Lakes, NJ). A minimum of 300,000 events were collected for each sample. Results represent the percent of cell expressing the surface markers.

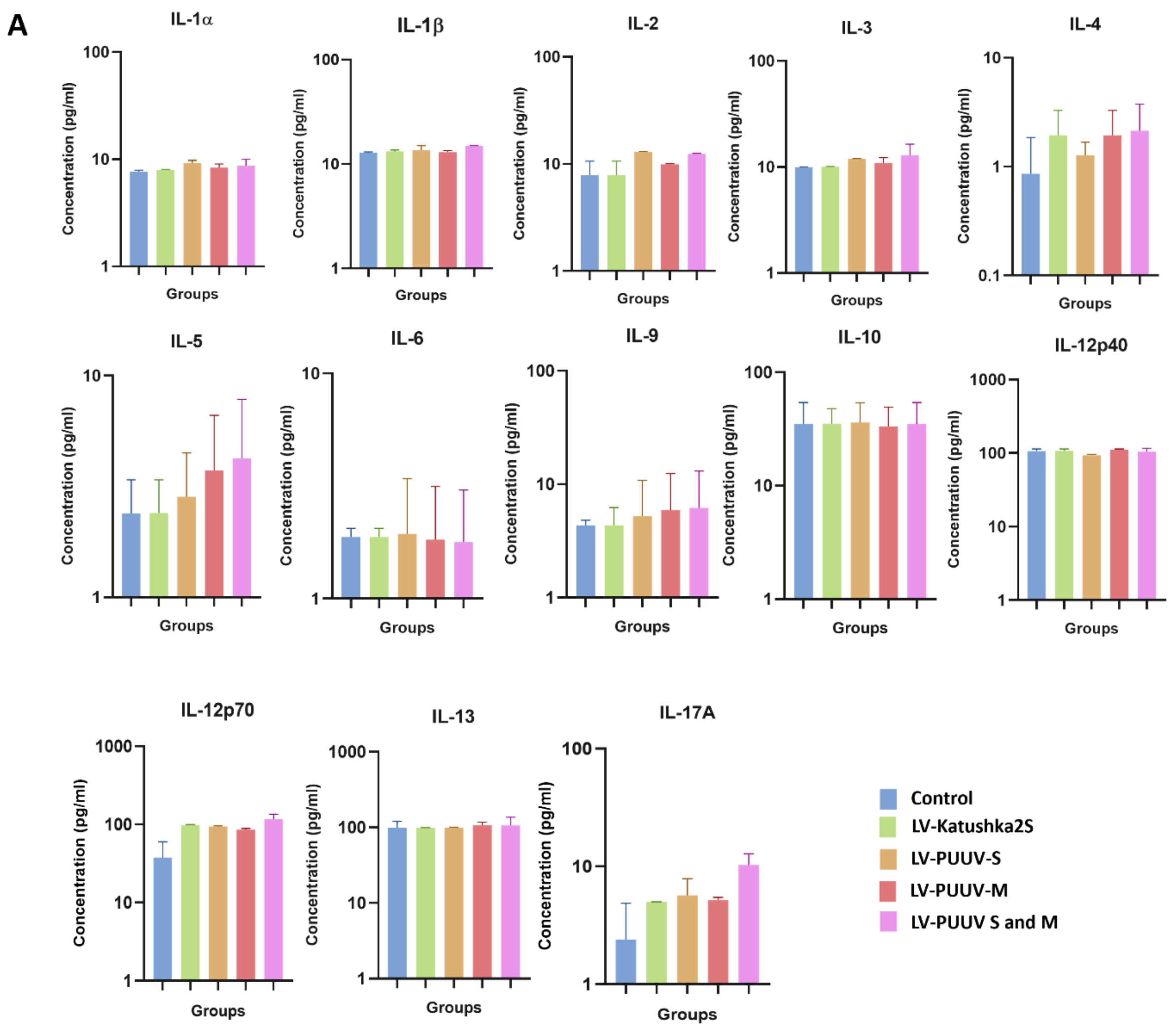
A
IL-1α
IL-1β
IL-2
IL-3
IL-4
IL-5
IL-6
IL-9
IL-10
IL-12p40
IL-12p70
IL-13
IL-17A
Concentration (pg/ml)
Groups
Control
LV-Katushka2S
LV-PUUV-S
LV-PUUV-M
LV-PUUV S and M

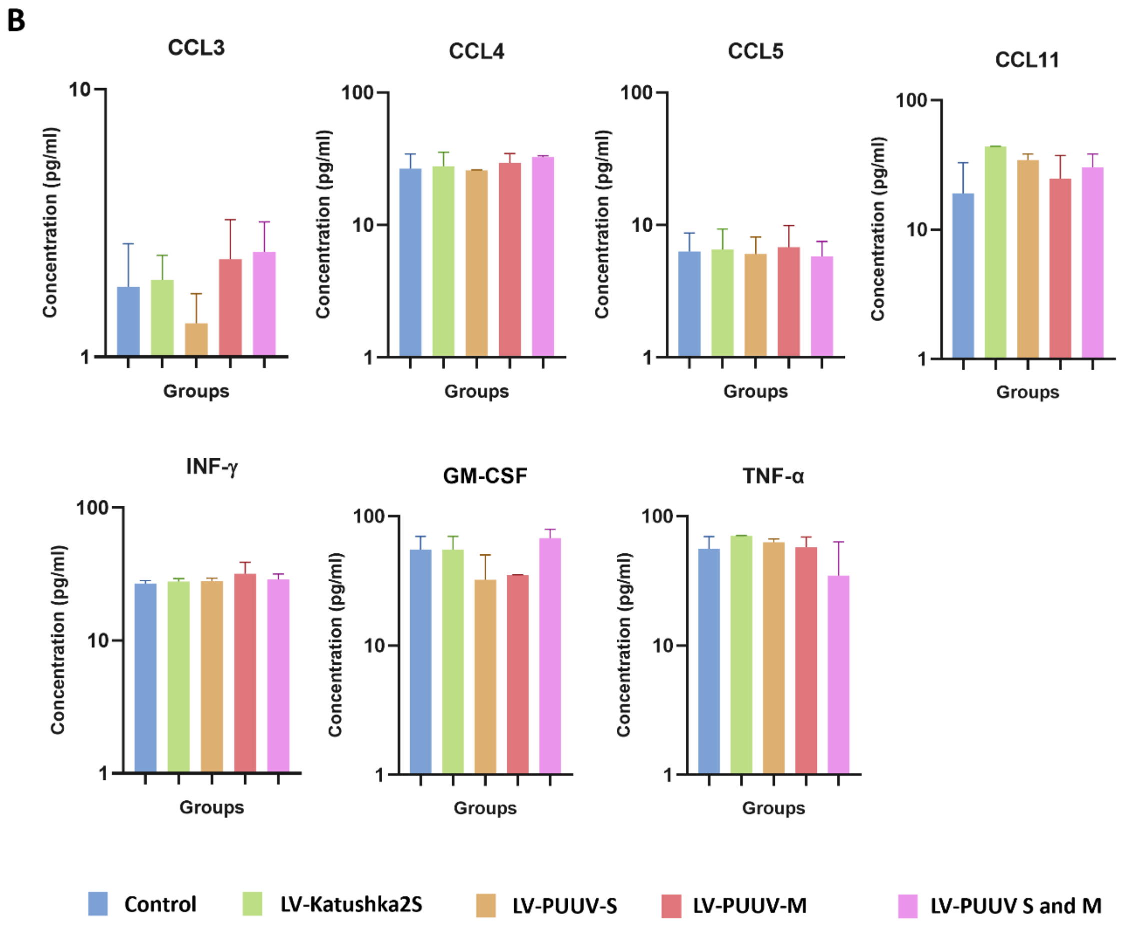

Figure S2. Cytokine and chemokine levels in mMSCs supernatant. mMSCs were transduced with LV-PUUV-S, LV-PUUV-M as well as combined LV-PUUV-S and PUUV-M lentiviruses. Supernatants from non-transduced as well as cells transduced with LV-Katushka2S were used as control. (**A**) Interleukin levels; (**B**) cytokine and chemokine levels. Data is represented as median ±SD. * $p < 0.05$, ** $p < 0.01$, *** $p < 0.005$, **** $p < 0.0001$. p value < 0.05 was considered statistically significant.

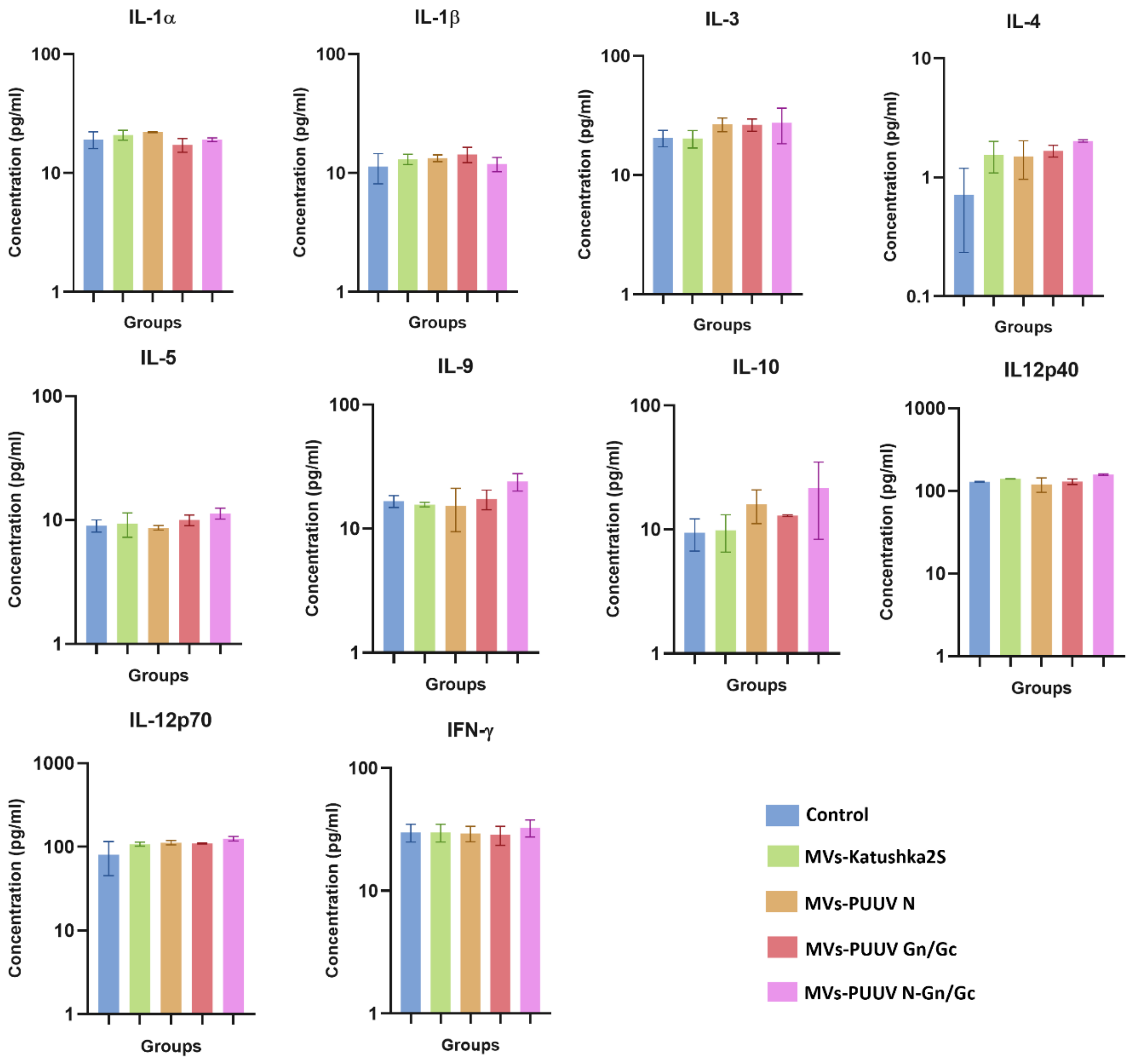

Figure S3. Cytokines and chemokine analysis of MVs cargo. Cytokine and chemokine levels were analyzed in MVs carrying PUUV N, PUUV Gn/Gc and combined PUUV N and Gn/Gc proteins, using Multiplex analysis. MVs generated from non-transduced as well as transduced with LV-Katushka2S mMSCs were used as control. MVs (50 µL in each well) with total protein (10 µg) were loaded into the well. Data is represented as median ±SD. * $p < 0.05$, ** $p < 0.01$, *** $p < 0.005$, **** $p < 0.0001$. p-value < 0.05 was considered statistically significant.

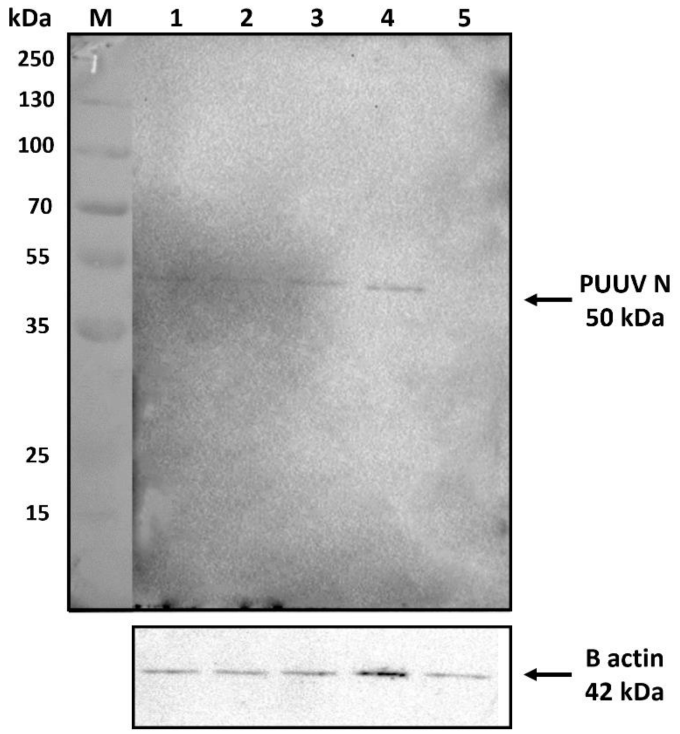

Figure S4. PUUV N proteins in A549 cells after treatment with different concentrations of MVs. Proteins (10 μg) were loaded in each well and separated using electrophoresis. Primary rabbit anti-N protein were used to detect PUUV N. PUUV proteins were visualized using Clarity ECL Substrate solution. PUUV N (50 kDa) protein in A549 cells treated with 10μg, 15 μg, 20 μg, 30 μg of MVs carrying PUUV N (lane 1,2,3,4, respectively). A549 cells treated with MVs from non-transduced MSCs, were used as control (lane 5).

A1

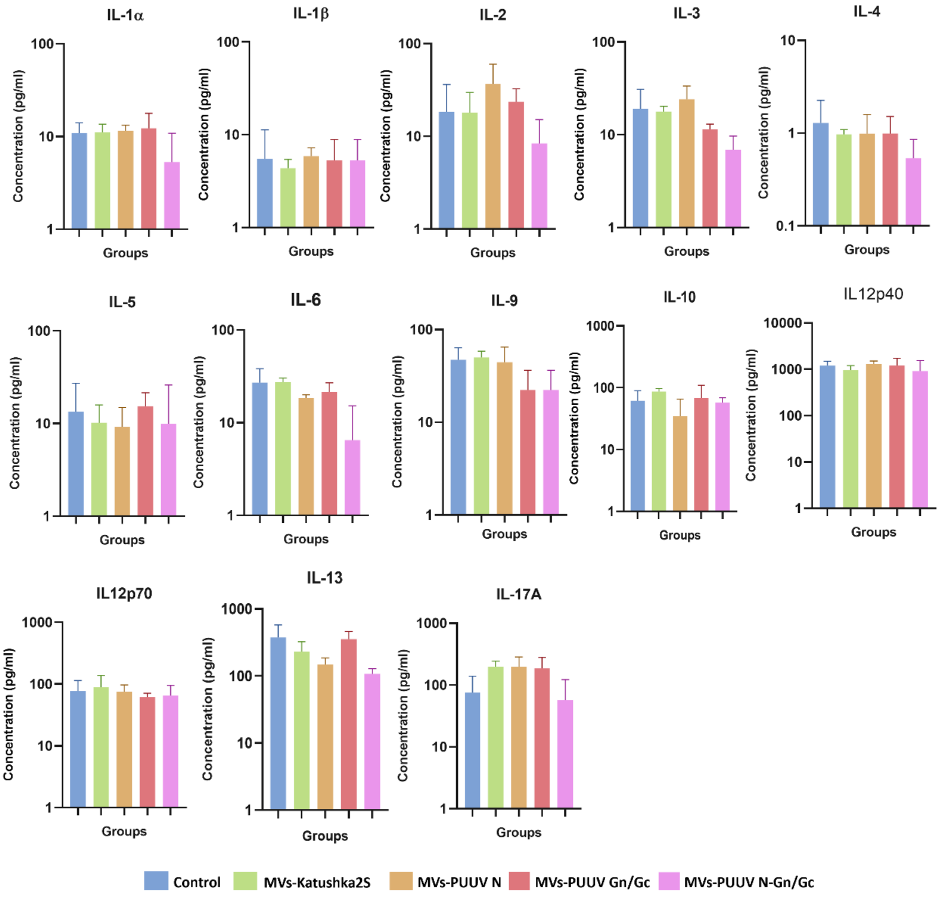
IL-1α
IL-1β
IL-2
IL-3
IL-4
IL-5
IL-6
IL-9
IL-10
IL12p40
IL12p70
IL-13
IL-17A
Concentration (pg/ml)
Groups
Control
MVs-Katushka2S
MVs-PUUV N
MVs-PUUV Gn/Gc
MVs-PUUV N-Gn/Gc

A2

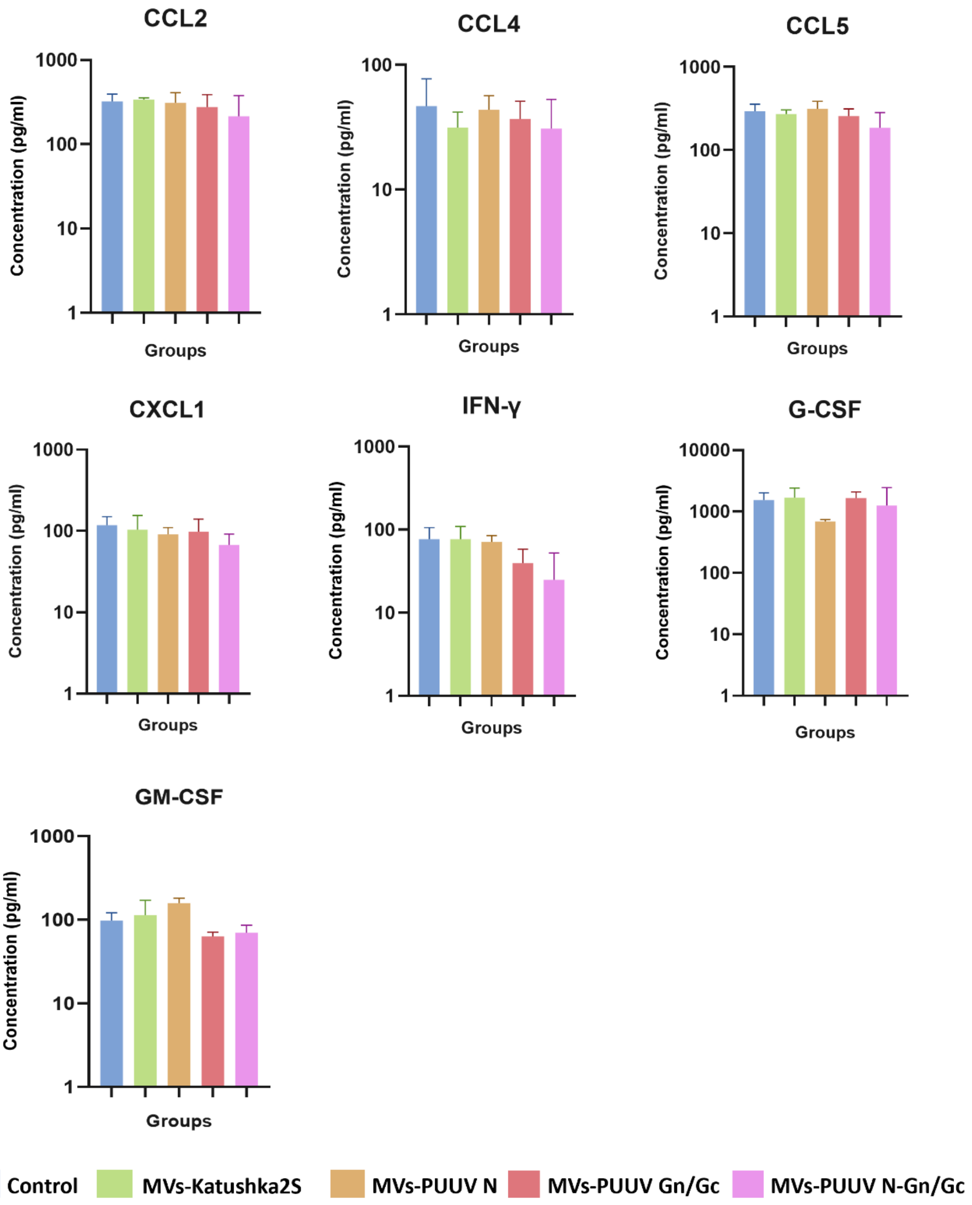
CCL2
CCL4
CCL5
CXCL1
IFN-γ
G-CSF
GM-CSF
Concentration (pg/ml)
Groups
1
10
100
1000
10000
Control
MVs-Katushka2S
MVs-PUUV N
MVs-PUUV Gn/Gc
MVs-PUUV N-Gn/Gc

B1

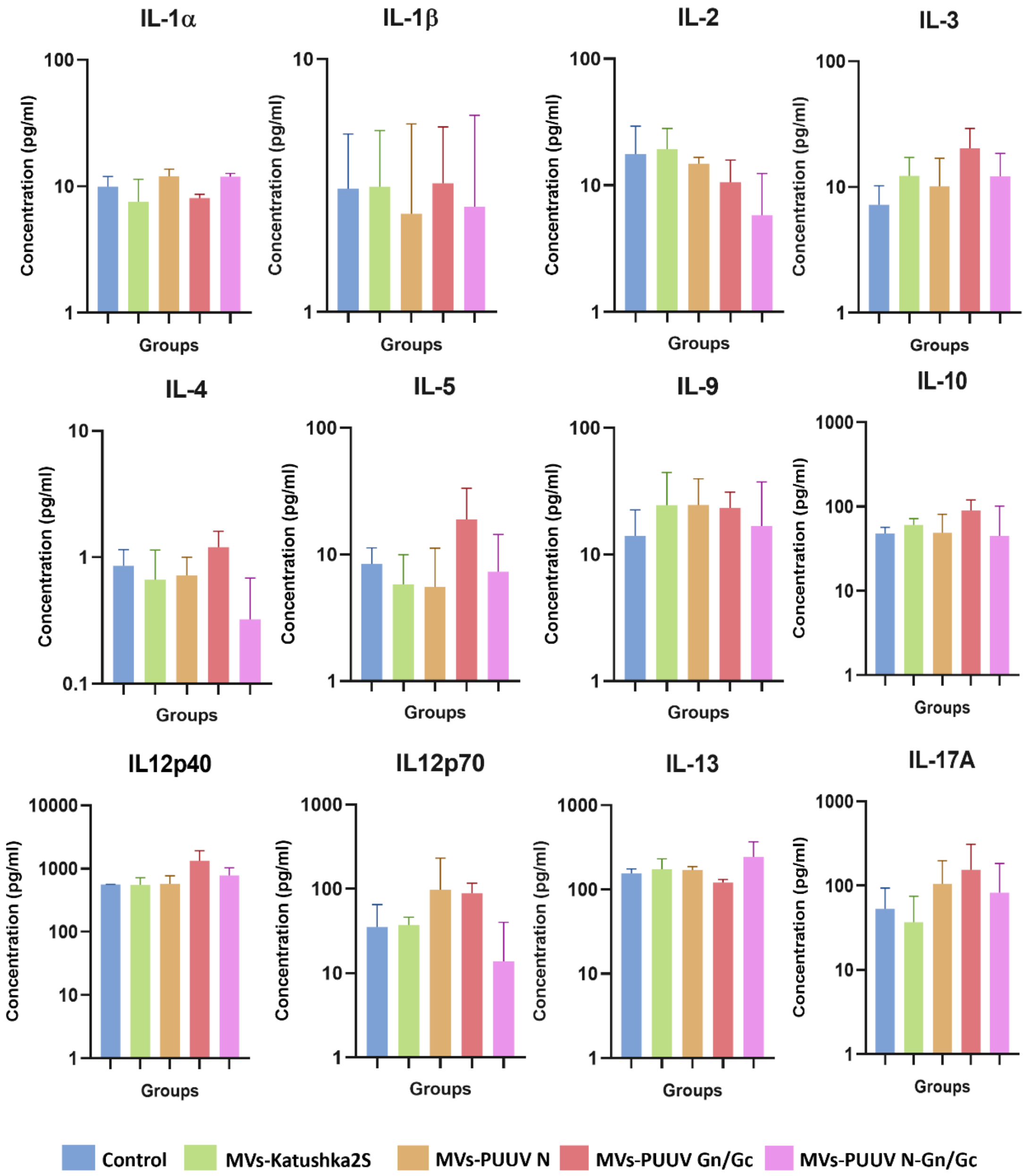
IL-1α
IL-1β
IL-2
IL-3
IL-4
IL-5
IL-9
IL-10
IL12p40
IL12p70
IL-13
IL-17A
Concentration (pg/ml)
Groups
Control
MVs-Katushka2S
MVs-PUUV N
MVs-PUUV Gn/Gc
MVs-PUUV N-Gn/Gc

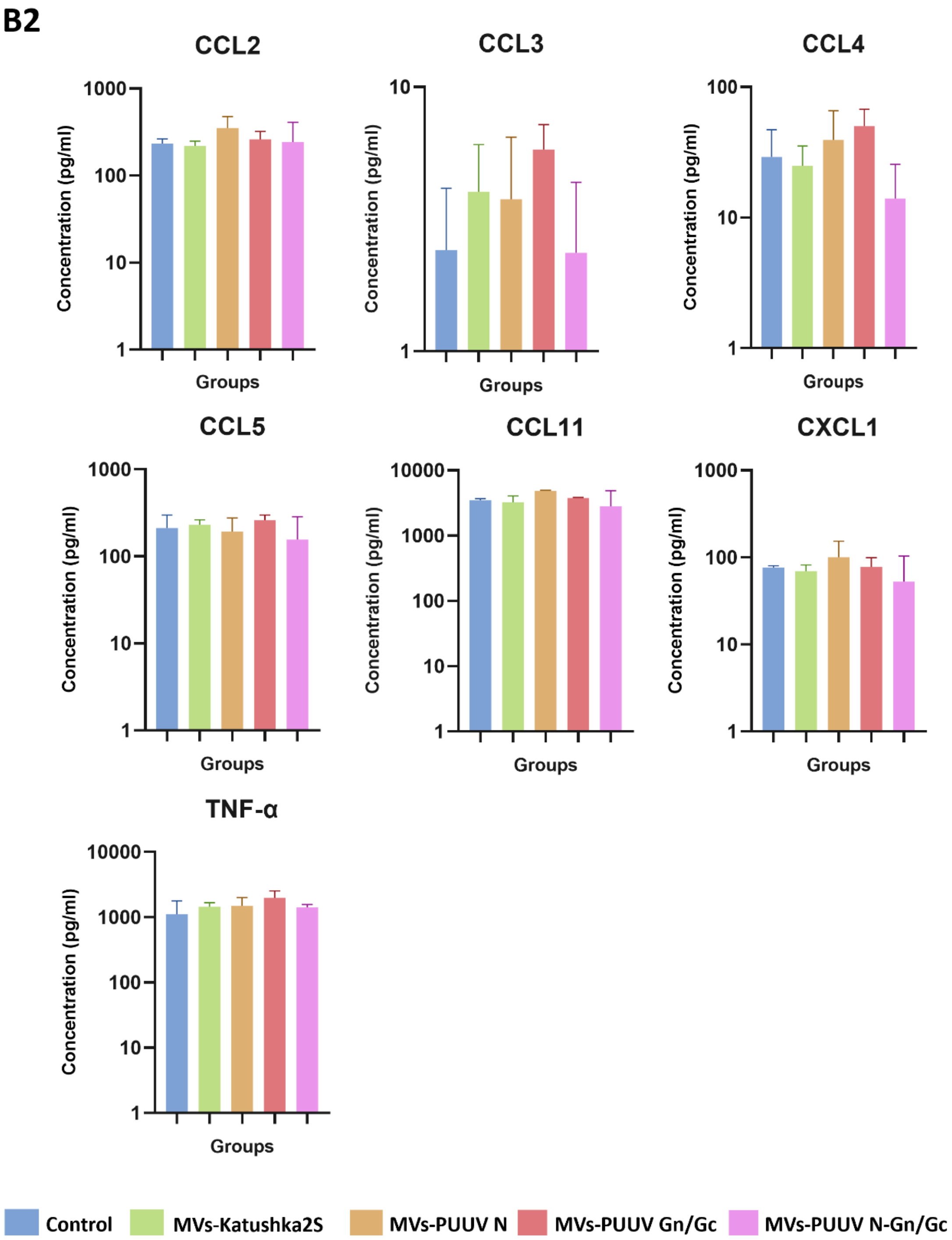

Figure S5. Serum cytokine analysis in mice treated with MVs at 14 and 28 days. Serum level of cytokines and chemokines was determined by using Multiplex method (BioRad). Serum was collected from mice treated with MVs containing PUUV N and Gn/Gc proteins, as well as their combination. Serum from mice treated with 0.9% NaCl solution as well as from mice treated with MVs-Katushka2S served as control. (**A1**) Interleukin levels 14 days after MVs treatment, (**A2**) cytokine and chemokine levels 14 days after MVs treatment; (**B**) Serum cytokines and chemokines levels 28 days after MVs treatment. (**B1**) Interleukin levels 14 days after MVs treatment, (**B2**) cytokine and chemokine levels 14 days after MVs treatment. Data is represented as median ±SD. * $p < 0.05$, ** $p < 0.01$, *** $p < 0.005$, **** $p < 0.0001$. p value < 0.05 was considered statistically significant.
